# Supplementary material for: Are carbon emissions trading and green financial instruments synergistic? -Comprehensive quantitative research based on content analysis
Source: PLoS One. 2024 Mar 7;19(3):e0298601. doi: 10.1371/journal.pone.0298601 (PMC10919601; doi:10.1371/journal.pone.0298601)
Supplement: S1 Data — (ZIP) [file pone.0298601.s001.zip › Data/地级市Z_F工作报告.zip!/║┌┴·╜¡/║┌║╙╩╨/└┤╫╘╣1⁄2╓┌║┼í╛┬φ┐╦╩2╛▌═°í┐_2009.docx]

各位代表：

现在，我代表市人民政府向大会作工作报告，请审议，并请列席会议的同志提出意见。

一、2008年工作回顾

过去的一年，政府工作在省委、省政府和市委的正确领导下，深入贯彻落实科学发展观，按照市委四届五次全会总体部署，紧紧围绕市四届人大二次会议确定的目标任务，全力实施“十项绩效工程”和“五个一”活动，积极培育“四型经济”，经济社会继续保持良好发展态势。

——经济实现较快发展，综合实力明显增强。预计全市生产总值206亿元，增长15.2%。三次产业分别增长18%、20%和11.4%。城镇固定资产投资54.4亿元，增长39.5%。社会消费品零售总额39.6亿元，增长19%。对外贸易进出口总值28.5亿美元，增长20.8%。全市全口径财政收入13.2亿元，一般预算收入7.2亿元，分别增长46.1%和52.2%；市直全口径财政收入3.8亿元，一般预算收入1.8亿元，分别增长33.7%和38.1%。爱辉、逊克财政收入步入亿元行列。城镇居民人均可支配收入8177元，农村居民人均纯收入4845元，分别增长19%和22.8%。万元地区生产总值能耗下降5%。全市经济发展连续跨越新台阶，主要指标均创历史最好水平。

——粮食产能增势强劲，农业大市作用凸显。预计农业增加值50.1亿元，增长20%。粮豆薯总产202.3万吨，增长32.8%，为历年之最。其中爱辉增长59.2%，嫩江增长45.9%。实施农技、农机和工程“三项措施”，测土配方施肥等技术普遍推广，农场模式化栽培624万亩。秋整地面积950万亩，其中深松整地516万亩。土地规模经营面积480万亩。绿色有机食品监测面积350万亩。组建农机合作社50个，三年组建规划顺利完成。人工影响天气工程加快推进，爱辉富拉尔基、嫩江青年、北安石远等水库除险加固完工。“两牛一羊一鹅”和特色养殖规模不断扩大，畜牧业增加值6.5亿元，增长16.1%。林业产业产值增长33%，林权改革正式启动。转移农村劳动力17.6万人。开发境外土地75万亩。农资供应主渠道作用发挥良好，各类农村合作组织长足发展。新农村省级试点村达到83个，首批68个全部通过验收。场县共建在产业化合作、农业保险等方面取得新进展。

——工业经济快速增长，质量效益同步提升。预计全市规模以上工业增加值12.1亿元，增长42.9%。地方规模以上工业增加值10.3亿元，增长53.7%；利税2.8亿元，增长39.7%，提前两年完成“十一五”规划目标。各县（市）区工业都呈现强劲增长态势，其中爱辉规模以上工业增加值增长171.2%，北安增长140.6%。电力、煤炭、冶金等重点行业比重达80%，为工业快发展奠定了扎实基础。运行监控进一步加强，工业经济效益综合指数122.5。黑河热电厂、恒基水泥等十户重点考核企业综合能耗下降8%。园区集聚效应逐步显现，五秀山俄电工业区耗电8.3亿度，工业硅产量5.9万吨，分别增长61.7%和58.1%，上缴税金4188万元，增长158%。

——开放程度显著提高，招商引资成果丰硕。“两国一城”建设扎实推进，合作交流机制进一步完善。赴俄克拉斯诺亚尔斯克、雅库茨克推介会和黑河中俄项目联合推介会成功举办，合作领域不断向俄腹地延伸。外经贸运行质量明显提高，税收7097万元，增长53.6%。境内外加工基地加快推进，果蔬、汽车、工程机械出口规模不断扩大，在俄建设水泥粉磨站等项目进展顺利。新货运口岸开通，旅检大厅改扩建工程竣工，口岸出入境139.9万人次，增长10.2%；过货量33.8万吨。通过实施对口、登门、集中推介、驻外招商等举措，共落实招商项目162个，其中亿元以上项目19个。内资实际到位资金42.1亿元，增长46.7%；外资4106万美元，增长124.4%。对外宣传力度加大，发展环境日益优化。增加了进哈列车，开通了北京航线。黑河荣获中国魅力中小城市称号，五大连池入选中国最具投资潜力中小城市二十强。

——“四型经济”取得突破，消费拉动初见成效。大力发展旅游、试车、会议、度假经济，吸引力和影响力不断提高。中国优秀旅游城市创建活动稳步推进，接待国内外旅游者171万人次，其中边境游49万人次，分别增长35.2%和10.1%；旅游总收入19.6亿元，增长21%。五大连池风景区接待旅游者突破百万大关，增长42.8%。爱辉森林公园、《闯关东》影视文化旅游区正式开放，中俄民族风情园俄罗斯风情街主体完工，瑷珲历史陈列馆晋升为国家一级馆，孙吴二战遗迹、爱辉新生民俗风情旅游区和卧牛湖风景区建设加快，黑龙江知青博物馆、北方明珠塔等项目和五大连池风景区申遗、晋级工作顺利推进，嫩江农场跻身国家农业旅游示范区。来黑河试车企业和研发机构60家，消费收入2000万元。承办各级各类会议105个，比上年翻一番。度假和购物环境进一步改善。大黑河岛自由贸易广场投入运营。市区批发业、零售业、住宿餐饮业零售额分别增长21%、17%和19%。

——财税工作不断强化，保障水平日益提升。财源建设成效显著，一些项目进入回报期。煤炭、建筑行业税收监管得到加强，其中煤炭税收1.2亿元，增长92%。部门预算制全面推行，公务卡系统开始试点，工程项目在全省率先纳入政府采购。市直按月人均标准300元兑现了津补贴政策，行政事业单位职工住房公积金财政匹配比例提高到8%。大项目前期、新农村建设、招商引资、对外宣传、公益事业和民生工程投入不断加大。全市金融机构各项存款余额275.5亿元，比年初增长23.3%；各项贷款余额95.4亿元，比年初增长1.4%。

——城乡建设步伐加快，市容村貌明显改观。全市城建基础设施投入6.1亿元，其中市区2.1亿元。市区和各县（市）新一轮城市总体规划、重点区域控详规划开始编制。市区道路、绿化、亮化、建筑物立面装饰、小区庭院改造、黑龙江护栏装饰等工程竣工，城乡环境整治效果明显。嫩江污水处理厂、北安铁南休闲广场、逊克江畔公园扩建、孙吴红旗街延伸、五大连池给排水管网改造均已完工。全市棚户区改造投入8.4亿元，建设回迁楼58.7万平方米。城镇新建住宅83.3万平方米，人均住房建筑面积24平方米。农村泥草房改造9800户，住房砖瓦化率65.2%，人均住房建筑面积19.1平方米。解决了1.2万人饮水安全问题。建成农村公路1807公里，建设里程再创新高。生态市建设得到加强，城市空气质量二级天数达360天。

——经济社会统筹推进，各项事业协调发展。实施国家和省级科技项目22项，省级硅基新材料产业基地成立。招聘重点院校毕业生107名，其中市直50名。小学和初中辍学率分别控制在0.15%和0.97%，高中阶段毛入学率55.2%，素质教育稳步推进。五大连池和孙吴医院、14个乡镇卫生院改扩建完工，市中医院搬迁扩建列入国家支持项目。社区卫生服务覆盖面100%。市区和五大连池成功创建省级卫生城市。群众体育日趋活跃，获得省级以上体育比赛奖牌69枚，市青少年活动中心、群众文化活动中心和老干部活动中心投入使用。市广播电视中心和发射塔开工建设，数字电视开通。人口自然增长率-0.4‰。黑河地震台网监测中心建成。民族宗教、侨务、妇女儿童、残疾人、老龄、人防、档案、地方史志等工作取得新成绩。

——和谐黑河稳步推进，社会秩序安全稳定。全市新增城镇就业2.1万人，完成省下达指标的115%，登记失业率3.8%，2057户零就业家庭全部实现就业。职工工资和三条保障线资金发放率均为100%。城乡救助体系更加完善，涵盖基本生活、就业、教育、医疗等9个方面。老年福利机构达到41所，市流浪未成年人救助保护中心和儿童福利院列入国家支持项目。关心低收入群体和残疾人生活，设立平价商店3家。信访工作不断加强，重大疑难信访问题得到有效化解。安全生产形势稳定，共发生各类事故212起，下降28.6 %，死亡人数上升3.8%，经济损失下降34.3%，其中煤炭工业事故为零。森林管护面积50万公顷，卧都河及麦海移动航站完工，新增森警390名，连续五个防期没有发生重特大森林火灾。文明城市创建活动深入开展，市民素质明显提高。社会治安综合治理全面加强，命案发案量下降31.8%，现行命案破案率93%，“平安黑河”建设取得成效。圆满完成奥运安保任务。互贸区管理进一步强化，边境秩序安全稳定。

——自身建设得到加强，行政效能有所提高。办理人大代表、政协委员建议和提案157件，办结率100%。“十项绩效工程”全面启动，“五个一”活动取得初步成果。民主法制建设有效推进，依法行政水平有新提高。创办了政府公报，整合了政务网络资源，建立了电子查询系统。简化基本建设项目等服务流程11项，明确了政府各部门办结时限。人力资源和社会保障服务大厅、住房公积金审批办事大厅投入使用。应急机制初步建立，救援能力得到提升。完善了重大决策合法性审查及评估反馈、专家咨询论证、市民听证等决策机制，出台了行政问责暂行办法。公务员绩效考核及政务督查不断强化，党风廉政建设工作进一步加强。

2008年，我们坚持执政为民，践行承诺。18项重点工程扎实推进。除金湾水电站因投资方资金问题未按计划实施外，关鸟河水泥厂、海澜热电厂、黑河至呼玛公路、嫩江至多宝山公路以及城市建设项目完工，多宝山铜矿、翠宏山铁多金属矿加快建设，争光岩金矿、30万吨电石、国家大型商品粮基地项目顺利开工，俄电工业区、北安工业园区基础设施进一步完善，三吉屯120万千瓦煤电一体化、阿州—黑河油品储运、五大连池矿泉水扩能改造、黑河至嫩江公路前期工作取得进展，黑龙江大桥俄方前期工作有新突破。12项改善民生行动深入实施。黑河一中投入使用。“双创”活动深入开展。新型农村合作医疗农民参合率93.6%，补助标准提高到80元。市第一人民医院门诊大楼主体工程完工。城市低保月人均补差由102元提高到144元，农村年人均补差由351元提高到624元。市区生活垃圾处理场投入使用，污水处理工程开工。南大沟防洪综合治理工程完工。南出口路拓宽工程竣工，环城西路拓宽工程拆迁基本完成。廉租房租赁补贴受益12356户，实物配租房受益145户。东西部棚户区新建回迁楼10万平方米。市区新增供热能力200万平方米。建成治安防范视频监控点120个。

2008年，各级政府积极创新，破解难题，全市人民共同努力，我们较好地完成了年初确定的各项经济和社会发展目标。

各位代表，过去一年的发展和进步，是在金融危机影响不断加深、各种困难并存的情况下取得的，成绩来之不易，是省委、省政府和市委正确领导的结果，是人大、政协全力支持和推动的结果，是全市人民团结一致、共同奋斗的结果。在此，我代表市政府，向辛勤工作在各行各业的全市人民，向给予政府工作大力支持的各级人大代表、政协委员和各民主党派、工商联、无党派人士、人民团体，向关心支持地方工作的驻黑中省直单位和人民解放军、武警官兵，表示崇高的敬意和衷心的感谢！

在肯定成绩的同时，我们也清醒地认识到，全市经济社会发展仍存在不少问题，前进路上面临诸多挑战。主要表现在：金融危机影响不断加重，加快发展面临更加严峻的形势；受进口廉价转基因大豆冲击，农民增收受到一定影响；国内外进出口需求减少和俄方取消布市木材报关权，对进出口贸易产生不利影响；主要矿产品价格大幅下跌，影响新上矿产资源开发项目进度；部分企业产品增值幅度下降，增收难度加大；民生投入与群众期望还有很大差距。政府工作也存在缺点和不足，主要是思想解放程度不够，工作创新和抓落实能力有待进一步提高，等等。对此，我们一定高度重视，在今后工作中努力克服和解决。

二、2009年工作目标和总体要求

今年是我市实现“十一五”规划目标的重要一年，也是在重要战略机遇期实现突破发展的关键一年，需要我们正确认识形势，坚定发展信心。一方面，随着国际金融危机的影响进一步加深，我国经济运行中的困难增加，经济下行压力加大，我市经济也面临着不确定因素增多、有可能趋缓的严峻挑战。另一方面，中央实行积极的财政政策和适度宽松的货币政策，实施扩大内需、振兴产业的一揽子计划；我省加快推进东北亚经济贸易开发区、大小兴安岭生态功能保护区等“八大经济区”，实施千亿斤粮食产能、现代交通网络建设等“十项工程”，我们也面临着难得的发展机遇。总体看，挑战与机遇并存，机遇大于挑战。面对国际国内经济形势的发展变化，必须跳出黑河看黑河，着眼全局谋发展。

政府工作要坚持科学发展观，认真贯彻落实党的十七大、十七届三中全会和中央、省委经济工作会议精神，正确把握中央扩大内需促进增长政策，采取更加积极的措施应对危机，夯实现代农业发展基础，做好开发开放两篇文章，继续实施“十项绩效工程”，坚持产业发育与园区建设相依托，增加投资与扩大消费相促进，城市发展与农村发展相统筹，经济建设与社会建设相协调，进一步提升经济总量，优化经济结构，加快发展步伐，着力保障和改善民生，实现经济社会又好又快、更好更快发展。主要预期目标是，地区生产总值增长11%以上。城镇固定资产投资增长30%。社会消费品零售总额增长15%。对俄贸易保持稳定增长。全口径财政收入增长12%。城镇居民人均可支配收入增长8%，农村居民人均纯收入增长6%。万元地区生产总值能耗降低5%。

面对严峻的形势，要时刻保持清醒头脑，深入分析经济社会发展中可能遇到的困难和风险，把攻难点、解难题作为今年工作的基本要求，做好预防和化解的周密准备，把不利影响降到最低程度。要牢牢把握以下几个着力点。

——积极应对不断变化的形势，着力培育新的经济增长点。要密切关注国内外经济动态，及时调整工作思路，引进新企业，做强老企业，加快结构调整，提升产业层次，做到经济不减速，发展有后劲。

——认真贯彻中央推进农村改革发展决定，着力增加农民收入。要把促进粮食增产和农民增收作为农村工作的核心，尤其要抓好农民增收工作，想千方、设百计保证农民生活水平稳步提高。

——紧紧抓住拉动内需的政策机遇，着力扩大投资规模。要充分利用好国家拉动内需的各项政策，大力招商引资和吸引民间投资，加快经济和社会事业项目建设，扩张经济总量，保证投资高位增长。

——充分发挥中俄两个市场作用，着力拓展新的合作领域。要立足黑河地缘优势，重点发展对俄贸易，夯实平台基础，延伸辐射范围，努力建设国际性商品集散地和中转站、中俄原材料能源开发产业聚集地。

——不断打造消费热点，着力培育新型经济。要全力打造新的具有突出个性和较强吸引力的消费热点，进一步提高产业素质和服务水平，在更广的范围内聚集人流、扩大消费。

——努力深化改革，着力激发更大发展活力。要把改革创新作为保增长、促发展的关键，扎实推进财政、医疗、林权等重点领域改革和政府运行机制创新，进一步增强发展内在动力。

——突出改善民生，着力促进社会安定和谐。要把保障和改善民生放在更加突出的位置，采取更切合实际的措施，认真解决事关群众切身利益的问题，努力打造“平安黑河”、“和谐黑河”。

新的一年，我们遇到的困难和挑战是前进中的问题，既要把困难估计得更充分一些，把应对措施考虑得更周密一些，又要注重从变化的形势中把握难得的发展机遇和有利因素，以更加奋发昂扬的精神状态，更加求真务实的工作作风，攻坚克难，埋头苦干，力争今年工作在严峻形势下实现新突破。

三、抢抓机遇破解难题保持经济快速发展良好势头

科学发展观第一要义是发展。做好今年经济工作，对于维护我市改革发展稳定大局，具有十分重要的意义。

（一）积极推进“双增”工程，大力发展现代农业。努力促进粮食稳定增产和农民持续增收。农业增加值增长4%。实施标准化生产。开展丰产栽培模式推广年活动，抓好良种普及、测土配方施肥、节水灌溉和模式化栽培等节本增效技术应用。搞好农业示范基地建设，促进新技术、新品种引进和推广。加强农业信息、农产品质量安全检测和动物疫病防控等服务体系建设。建立淡季化肥储备制度，完善农村流通网络。抓好工程化建设。实施南部县（市）旱涝保收田规划，建设爱辉、五大连池节水灌溉示范区。开工逊克前进、北安海星和爱辉卧牛河水库除险加固工程。推进大黑河岛、逊克新鄂、孙吴胜利、爱辉双灯照等重要堤段护岸工程。做好农机化项目、农机购置和深松整地补贴争取工作，增加农机合作社数量，扩大规模和覆盖面，深松整地400万亩。完成人工影响天气工程。提高产业化水平。科学规划产业化发展布局，培育特色主导产业，实现集约发展。扶强孙吴联凯大豆、嫩江南华糖业和兴邦药业、爱辉山珍集团和北安完达山乳业等龙头企业，推进嫩江嵩天等一批新的产业化项目，提高主要农产品加工率。完善利益联结和产销对接机制，鼓励农企共建原料基地，发展新型农企联合体。促进规模化发展。按照依法自愿有偿原则，引导农民以各种形式规范流转土地承包经营权，发展专业大户、家庭农场和农民专业合作社。建立土地承包经营权流转市场，同步抓好农村富余劳动力向非农产业转移工作。完成大型商品粮生产基地建设工程，扩大大豆、小麦、甜菜、亚麻、马铃薯和北药等基地规模，建设北药示范带。大力发展浆果产业。加快奶牛、肉牛和大鹅产业带建设，扶持养殖大户和专业小区。建设黑龙江、五大连池特色水产养殖示范带（区）。发展农村合作经济组织，农民参加比率提高到25%以上。加快一体化步伐。拓宽共建领域，推进场乡共建、林乡共建、企乡共建和城乡共建。在农业产业化、小城镇建设和医疗、教育等方面实现区域资源共享。农机跨区作业320万亩。加强与中储粮油脂公司合作，建设大豆储备基地。推进新农村建设。巩固第一轮试点村成果，启动第二轮试点村建设。加快泥草房改造，新建人畜饮水工程90处，推进广播电视“村村通”工程。抓好嫩江科洛、北安赵光省级试点乡镇建设。做好老区建设和扶贫开发工作。

（二）突出重点项目建设，促进投资持续增长。把投资作为拉动经济发展的主导力量，全力实施51项重点项目。推进重点产业项目。建设多宝山铜矿、翠宏山铁多金属矿、争光和东安岩金矿、库南铅锌矿、金湾水电站、矿泉水开发、矿泉理疗中心、爱辉风能发电等项目。推进宝发、华昌煤矿新建项目。争取欧林、金三角煤矿竣工验收，同兴煤矿开工建设。做好二道坎、乌二水电站、嫩江和五大连池风能发电等项目前期。抓好公路项目建设。做好省管高速公路服务工作，加快市管干线公路建设。嫩黑公路215.3公里、加黑公路111公里、黑嘉公路66公里完成路基及桥涵主体工程。新建农村公路700公里。按时完成征地拆迁任务，解决资金缺口，保证公路建设顺利推进。加大招商引资力度。坚持科学招商，明确招商方向，改进招商方式，做到专业化、个性化、集约化相结合，提高招商工作质量。立足“长三角”、“珠三角”等发达地区，面向国内和世界500强企业，吸引产业、资本、技术转移。充分发挥驻外招商员作用，在重点地区聘请招商员。落实优惠政策，营造良好环境。

（三）加快提升产业层次，培育工业新优势。面对国内外市场变化，提高科技含量，加快发展速度，扩张工业总量，壮大总体实力。全市规模以上工业增加值增长16%。培育园区科技产业。加快五秀山、锦河、爱辉俄电工业区、逊克工业园区及黑河对俄进出口加工区建设，加强与国内外科研机构合作，注重科技成果引进转化。依托省级硅基材料研究中心，加快有机硅、多晶硅、碳化硼等硅基、硼基系列产品研发，促进产业升级。推进年产2000吨高纯度硅、14万吨工业硅、30万吨电石化工等续建项目。抓好北安工业园区高科技产业项目入园，推进嫩江工业园区动物制药等项目建设。延长矿产开发链条。加快推进国电集团煤电一体化项目，促进煤炭资源转化。抓好多宝山地区总体规划，科学有序开发矿产资源。推进多宝山电解铜、嫩江北泰铜业项目建设，开发电解铜及铜系列产品。抓好铁、钼等矿产开发项目，培育精深加工企业，提高产品附加值。加快企业改造升级。引进新技术、新设备、新工艺，支持建材、医药、食品、木材等企业技术改造。加快黑河盛泰建材、五大连池浸油、北安宜品乳业、孙吴大果沙棘等企业扩能改造和产品开发。加强运行监控。及时掌握煤、电、油等原材料和产品市场信息，综合运用调控措施，提高工业抗风险能力。开展企业增信活动，帮助企业融资，减免行政规费，为企业运营创造条件。

（四）提高经贸合作效益，提升对外开放水平。以黑河为节点，继续推进“两国一城”建设，扩大合作圈，调整贸易结构，建设连接国内国外两个市场的物流通道，打造服务全国、辐射俄罗斯的商品集散地。增加口岸过货量。根据国家边境小额贸易政策变化，研究制定相关措施，扶持稳定本港过货企业，引进一批经贸主体，帮助争取进出口专营权，解决贷款、运输等困难。调整优化贸易结构，扩大进出口规模。协调俄方恢复布市港木材报关权。过货量比上年有所增长。扩大地产品出口。开展地产品市场调研，制定兑现奖励政策，继续扩大木制品、红砖、蔬菜等地产品出口。大力扶持黑河正兴磨料、嫩江辰鹰乳业、北安进发塑料等出口企业。协调布市口岸，增加恒基水泥冬季出口量。拓展合作范围。调整市场份额，巩固阿州市场，继续向克拉斯诺亚尔斯克、雅库茨克、涅留恩格里区等俄腹地延伸，加强在铁矿煤矿开发、蔬菜种植、汽车维修、毛皮贸易、钻石加工等方面合作。完善与俄远东地区工商会联络机制，不断拓展市场空间。建设经贸合作平台。启动保税港区和跨江经济合作区前期工作。推进爱辉、逊克、北安蔬菜出口基地建设，筹建中俄果蔬仓储库。扩大在俄工程承建、劳务合作、土地承包规模，完成石油炼化厂前期准备。建设境外木材、建材、石化等加工基地。举办大黑河岛商品展销会，继续与俄方联合开展推介活动。加强贸易通道建设。探索与阿州共建运输综合体，开辟集装箱国际运输通道。完成黑河港改扩建。发挥新货运口岸作用，建立市场竞争机制，降低通关成本。加大对俄协调力度，提高通关效率。敦促俄方做好黑龙江大桥建设前期工作。

（五）培育新兴消费热点，聚集人流扩大消费。加快发展“四型经济”，打造旅游名城、试车基地、会议之都、避暑天堂。完善服务设施。坚持政府主导和市场化运作，培育旅游支柱产业。接待国内外旅游者196万人次，增长15%。推进中俄民族风情园、瑷珲历史文化旅游区、五大连池风景区、孙吴二战遗迹等景区建设。做好五大连池风景区火山博物馆和黑龙江自然博物馆前期工作。加快火山、矿泉、民俗、冰雪、知青、红色等旅游文化资源和黑龙江岛屿开发。做好五大连池风景区申遗和5A级、瑷珲历史陈列馆4A级景区争取工作。加快五大连池风景区机场项目前期工作。加强与韩、日、德及国内汽车生产厂家和研发机构合作，建设寒带试车基地，增加规范的试车场数量。抓好高档商务酒店、星级宾馆、家庭公寓和林间度假村建设，完善商业网点和休闲设施。强化大黑河岛自由贸易广场功能，培育对区域外具有辐射力的市场。优化消费环境。突出特色鱼类、绿色山珍和俄式西餐，发展特色餐饮业。深度开发旅游纪念品，创出系列品牌。成立试车协会，搞好服务。开展服务行业等级评定，加强从业人员培训，提高服务水平。制定旅游业管理办法，建立健全维权组织，严厉打击非法导购、价格欺诈和境外赌博等行为。强化宣传促销。编制黑河大旅游圈规划，创建优秀旅游城市。强化市场开发，开辟新线路，实现主要景区景点互动。以启动异地办照试点为契机，加强与央视、省级媒体合作，继续扩大黑河城市形象和景区景点广告覆盖面。培育文化、体育产业，争取举办国家级大型文体活动，打造黑河山水风情和五大连池实景大型演出。邀请知名作家、影视剧组来黑河采风，创作有地域特色的文学、影视作品。积极申办各级各类会议，承办数量比上年翻一番。加大对俄宣传力度，吸引俄公民入境旅游、度假和购物。

（六）坚持保护开发同步，构筑良好生态屏障。启动实施生态功能区建设规划，积极推进生态市建设，加快修复和提升生态功能。加大森林资源保护力度。做好森林防火工作，力争不发生重特大森林火灾。抓住我省加快平原造林绿化契机，开展城镇、村屯和道路、河流两侧绿化，加快农田林网建设和退耕还林，全面完成省下达的各项造林工程。严厉打击毁林、毁草、毁湿开垦和乱砍滥伐等行为。推进爱辉区林权改革试点，做好其他县（市）改革准备。林业产业产值增长25%。改善生态环境。加强国家级生态屏障建设，做好嫩江、五大连池国家级生态示范区申报工作。按照整装勘查、系统规划、集约开发、稳步推进原则，加大探矿权管理和采矿权核查力度，规范开发秩序。鼓励企业开发有益组份，有效利用尾矿及废渣、废水。建立矿山地质环境评价制度，严格落实生态恢复责任。强化国土保护，推进土地开发复垦项目和逊克国土防护工程，抓好孙吴黑土流失区及讷漠尔、乌裕尔、逊别拉河等流域治理。抓好节能减排工作。严格执行“三同时”制度，严把建设项目环保关。抓好主要污染物削减工作，黑河、北安和五大连池污水处理厂投入使用，完善黑河、北安和嫩江热电厂脱硫配套设施。完成重点污染源监控工程建设。淘汰落后产能，关闭环保不达标企业。化学需氧量下降2%，二氧化硫排放量下降0.5%。

（七）做好财政金融工作，支持经济社会发展。加强财源建设，优化金融环境，为经济社会发展提供强有力支撑。促进财政增收。积极扶持加工企业和创税大户，巩固壮大税源基础。坚持依法治税，抓好煤炭、建筑、餐饮等重点行业税收征管，确保应收尽收。争取更多项目纳入省扶持范围，培植新兴和后续财源。出台规范政府各类收费管理的意见。加强财政监管。继续推行国库集中支付制度，抓好财政支出绩效评价试点。加强工程项目政府采购管理和财政投资项目评审工作。建立健全国有资产监管机制，抓好国有资产动态管理及整合、置换和变现。推进金财工程建设，保证财政资金安全。强化重点项目、重点资金审计监督，提高财政资金使用绩效。扩大公务卡改革试点，规范公务支出。优化支出结构。强化预算约束，严格控制弹性支出，从紧安排一般支出，保证重点支出，集中支持大项目前期、公益事业和民生工程，适度增加教育、卫生、就业、稳定等方面投入。提高市直部门经费保障水平，人均增加500元。加大向上争取力度。抓住国家扩大内需、加大投资机遇，积极沟通对接，建立信息捕捉和通报机制，做好项目编制、论证和申报工作，把符合国家政策的项目和资金争取到位，力争更多项目列入国家扶持计划。改善金融服务环境。完善政银企沟通协调机制，搭建合作平台。推进信用体系建设，强化企业信用评价、监督、管理。支持金融机构抓住国家信贷政策调整契机，拓展经营领域，扩大信贷规模，服务地方经济。

四、扎实推进城市建设和社会建设努力改善民生

科学发展观核心是以人为本。城市整洁优美，社会和谐稳定，群众安居乐业，是广大人民的共同愿望，也是政府工作的重要任务。

（一）提升城市品位，打造特色鲜明景观城市。按照“高起点、高品位、有特色”原则，准确把握功能定位和发展方向，彰显滨水生态园林城市风格和特色建筑文化魅力。科学规划好城市。始终把规划作为城市建设、管理和发展的首要工作，增强科学性、前瞻性和权威性。加快市区总体规划修编和控详规划、建详规划编制。聘请专家进行城市发展定位研究和城市风貌特色规划设计。指导各县（市）完成总体规划制定和控详规划编制。各县（市）要全面完成城市风貌设计。建立完善规划决策机制，开展规划执行监察，坚决制止和查处违规建设行为。精心建设好城市。市区遵循“西改、东扩、南调、北优”城市空间发展方向，打造街路“一轴十线”重点景观。继续推进棚户区改造，东部棚户区完成回迁楼建设，启动中央东大街西南侧土地开发项目；西部棚户区抓好拆迁安置、回迁楼以及保障性住房建设。建设回迁楼4.6万平方米。加快推进市区道路建设项目，铁路街拓宽，龙科街延伸，开发路中央街至王肃街段新建，通江路、王肃街沥青罩面等，“十一”前全部完工。抓好巷道、小区内的“硬覆盖”和“绿覆盖”工作，逐渐消灭裸露地。开展“万棵果树进社区”活动。建设大黑河岛步行栈道桥、第二交通桥、北方明珠塔。增加黑龙江公园雕塑。北安启动庆华棚户区改造工程，加快滨水公园建设。逊克完成口岸路收尾工程。风景区加快景点、矿泉带内棚户区和违章建筑拆迁工作。五大连池垃圾处理场开工，孙吴、嫩江垃圾处理场投入使用。严格执行招投标制度，加强监督管理，确保工程质量。严格管理好城市。坚持市场化、规范化、精细化、人性化、群众化原则，全面提高管理水平。对环卫作业、清扫保洁、道路维修、照明管护、绿化养护等全面实施市场运作，公开招标。完善城市管理规章体系，使每项管理都有法可依、有章可循。提高标准，细化指标，做到城市管理全覆盖，无死角。遵循便民利民原则，科学规划和建设农贸市场、停车位、指示牌、盲道、公厕等设施。强化宣传教育，增强群众爱护城市意识，调动机关事业单位、商家和社会各方面力量，形成城市管理合力。

（二）加强社会建设，促进经济社会协调发展。坚持以人为本，努力办好与人民群众切身利益密切相关的各项社会事业。高度重视人才工作。落实人才政策，提高人才素质，改善人才结构。留住和用好现有人才，抓好学科带头人梯队建设。做好事业单位改革准备，建立人才激励机制。编制需求目录，重点引进30名研究生以上学历人才，以及个别行业急需重点本科院校毕业生。强化科技支撑能力。加强对俄科技交流合作中心建设，建立中小企业对俄合作服务平台。强化先进技术引进吸收和再创新，建设俄罗斯高纬寒地经济树种繁育基地，力争辟建国家级技术创新暨TRIZ理论基地。办好人民满意教育。巩固发展义务教育，着力推进普通高中课改，加快发展职业教育。全面实施素质教育，抓好学生综合素质评价体系建设。发展特色教育，打造全国知名俄语教育品牌。抓好乡镇中心校建设和改造。全面实行中小学校长竞聘上岗制度，进一步改善办学条件，努力提高教育质量。新建市特殊教育学校。支持黑河学院基础设施建设。提高医疗服务质量。做好医改准备，保证公共医疗卫生公益性。加强农村三级卫生服务网络和城市社区卫生服务体系建设，调整市区卫生服务中心布局。新建和改造16个乡镇卫生院。抓好食品药品监督和重大传染病预防控制工作。巩固新型农村合作医疗成果，实施“一证通”制度，参合农民自主选择定点医院。深入开展中俄医学交流合作，建设中俄国际康复保健中心。市第一人民医院门诊大楼、北安第一人民医院病房楼投入使用，黑河、嫩江中医院搬迁新建工程开工。做好人口与计划生育工作，人口自然增长率控制在4.8‰以内。统筹发展其他事业。深入开展城乡群众文化活动，成立21个乡镇文化站。组织好建国60周年文艺演出，举办第二届农民艺术节。开展中俄文化交流，举办摄影展、巡回演出等。开展全民健身运动，发展竞技体育。举办全市第四届田径运动会。建设青少年冰上活动中心。加快有线电视数字化平台建设，市广电中心和发射塔投入使用。继续规范法律服务和法律援助工作。全面实施妇女、儿童发展规划，关心支持残疾人事业，落实老年人优待补贴政策。继续做好“双拥”工作。进一步发展民族宗教、侨务、防震减灾、人防、档案、地方史志等其他社会事业。

（三）推动和谐进程，保持社会安定有序。更加关注城乡弱势群体，在保增长、促发展的同时，努力改善民生，提高群众幸福指数。突出抓好就业再就业工作。面对严峻的就业压力，将促进就业和再就业作为民生之本，继续开展“双创”活动。扶持壮大非公有制经济，促进劳动密集型中小企业发展，创造更多就业岗位。提倡和鼓励弹性就业、自谋职业、自主创业。落实贷款支持、社会保险和工资补贴等优惠政策，抓好职业培训和就业服务。重点做好零就业家庭和就业困难人员安置工作。全市新增就业1.8万人，城镇登记失业率控制在4.6%以内。加强社会保障体系建设。完善城乡最低生活保障制度，实行动态管理、分类施保，将符合条件的贫困人口全部纳入保障范围。做好城镇居民医疗保险启动工作，建设社区工作平台。出台低收入家庭认定办法，落实特殊困难临时救助专项资金。廉租房实物配租150户，逐步解决低收入家庭住房问题。落实安全生产责任。全面推进企业安全质量标准化建设，切实搞好重点行业和领域专项整治工作，加强矿山、道路交通、消防、危险化学品和人群密集场所安全防范，避免各类重特大安全事故发生。加大信访工作力度。加强目标管理，落实领导责任制。整合行政首长信访电话，推进信访信息网络体系建设。加强矛盾纠纷排查和化解工作，提高初信初访和疑难信访案件化解率，降低重访、集体访和越级访发生率。推进维稳创安工程。强化社会治安综合治理，严格控制发案量，提高破案率。加大社区和行业场所、重点单位、要害部位治安管理和整治力度。继续实施“警灯闪烁”工程，建设视频监控系统，构建适应动态社会环境的治安防控体系。加强边境管理，确保边境安定。

五、切实加强政府自身建设

面对严峻复杂的经济形势和繁重紧迫的发展任务，我们必须坚决贯彻市委决策，主动接受人大依法监督和政协民主监督，进一步完善工作推进方式，加强服务型政府建设，积极、全面、正确地履行职能。提高科学发展能力。扎实开展好学习实践科学发展观活动，不断提高把握发展机遇、应对风险挑战的能力，不断提高领导科学发展、促进社会和谐的能力。创新工作思路和方法，坚持把科学发展观落实到解放思想、破解难题上，把学习成果体现在提高公务员队伍整体素质上，使科学发展观贯穿想问题、做决策、抓工作的全过程。提高工作落实能力。进一步创新运行机制，继续推进“十项绩效工程”，开展“五个一”活动。各县（市）区也要创造切实可行的抓落实载体。完善公务员绩效考核体系，落实问责制度，严厉治懒、治散、治庸，强化执行力。加强各部门协调配合，主动为报件单位提供优质快速服务。提高依法行政能力。加强政府内部监督和群众监督，严格遵守政府议事规则和重大事项集体决策、专家咨询论证、市民听证制度，确保行政权力依法公正行使。严格执行行政执法责任制和行政过错责任追究制，强化执法监督，推动政府各项工作法制化。继续组织实施“五五”普法活动。提高公共服务能力。做好新一轮行政管理体制改革工作。继续减少和规范行政审批，加快电子政务建设，推进网上办公。进一步扩大政务公开范围，提高政府工作透明度。完善应急救援快速反应机制，提高快速反应能力，确保及时处置各类突发事件，妥善解决群众困难。统筹政府资源，探索市区共建。提高廉洁从政能力。认真坚持党风廉政建设责任制，进一步健全和落实“一岗双责”制度。加强对人财物管理和使用的监督，坚决预防和惩治腐败。继续深入开展“双评”活动，大力纠正行业和部门不正之风，严肃整治损害群众利益和破坏投资环境行为。

各位代表，新的一年，我们面临着严峻挑战和前所未有的机遇。让我们更加紧密地团结起来，在省委、省政府和市委的正确领导下，坚持以发展为上、民生为本、务实为先、律己为诫，不辜负全市人民的重托，共同谱写黑河经济社会科学发展、和谐发展的新篇章！
